# Only the Lonely: A Study of Loneliness Among University Students in Norway

**DOI:** 10.32872/cpe.v2i1.2781

**Published:** 2020-03-31

**Authors:** Mari Hysing, Keith J. Petrie, Tormod Bøe, Kari Jussie Lønning, Børge Sivertsen

**Affiliations:** a Department of Psychosocial Science, Faculty of Psychology, University of Bergen, Bergen, Norway; b Department of Psychological Medicine, University of Auckland, Auckland, New Zealand; c The Norwegian Medical Association, Oslo, Norway; d The Student Welfare Organization of Oslo and Akershus (SiO), Oslo, Norway; e Department of Health Promotion, Norwegian Institute of Public Health, Bergen, Norway; f Department of Research & Innovation, Helse-Fonna HF, Haugesund, Norway; g Department of Mental Health, Norwegian University of Science and Technology, Trondheim, Norway; Philipps-University of Marburg, Marburg, Germany

**Keywords:** loneliness, students, young adults, partnership status, student accomodation

## Abstract

**Background:**

Loneliness is a major public health concern among college and university students, the evidence is inconsistent regarding whether there is an increasing trend or not. Furthermore, knowledge of the demographic determinants for loneliness are limited. The present study assesses recent trends of loneliness from 2014 to 2018, and explores demographic risk indicators of loneliness among students.

**Method:**

Data was drawn from two waves of a national student health survey from 2014 and 2018 for higher education in Norway (the SHoT-study). In 2018, all 162,512 fulltime students in Norway were invited to participate and 50,054 students (69.1% women) aged 18-35 years were included (response rate = 30.8%). Loneliness was measured by “The Three-Item Loneliness Scale” (T-ILS) and one item from the Hopkins Symptom Checklist-25 (HSCL-25).

**Results:**

Age showed a curvilinear association with loneliness, with the youngest and oldest students reporting the highest level of loneliness across all measures. Other significant demographic determinants of loneliness were being female, single and living alone. There was a considerable increase in loneliness from 2014 (16.5%) to 2018 (23.6%, p < .001), and the increase was particularly strong for males, for whom the proportion of feeling “extremely” lonely had more than doubled.

**Conclusion:**

The high rate of loneliness and the increasing trends indicate the need for preventive interventions in the student population.

## Abstract (highlights)

- Loneliness among Norwegian university students increased from 2014 to 2018, particularly for males.
- Students in transitional periods, both the youngest and oldest reported the most loneliness.
- Being single and living alone were risk factors for loneliness.

Loneliness reflects the subjective feeling of disconnectedness and not belonging, and is often characterized as “a perceived discrepancy between desired and actual social relationships” (Portnoy, 1983). Loneliness is associated with more health problems (Hayley et al., 2017), and has been linked to an increased mortality risk (Holt-Lunstad, Smith, Baker, Harris, & Stephenson, 2015). Loneliness has often been thought of as a concern that peaks in older age. However, recent evidence has shown that the developmental trajectory is more U-shaped, with young adults having the highest levels of loneliness (Luhmann & Hawkley, 2016), followed by a second peak in older age groups.

The transition from adolescence to young adulthood makes college and university students a particularly vulnerable group for feelings of loneliness (Diehl, Jansen, Ishchanova, & Hilger-Kolb, 2018). This may be related to developmental-specific risk factors, such as moving away from home and their local community, and re-establishing new social networks. Surprisingly, the epidemiology of loneliness in young people has received scant attention. Knowledge of risk indicators and vulnerable subgroups are important in order to promote preventive actions. If loneliness is limited to, or peaks at the actual transition from moving away from home, a decline in loneliness over time should be expected among more senior students, which has been demonstrated in a German University sample (Diehl et al., 2018).

A recent UK study of 18-year-old twins found that loneliness was equally common across sexes and socioeconomic status (SES) (Matthews et al., 2019), whereas others have found both higher (Mounts, 2004) and lower (McWhirter, 1997) levels of loneliness among men. This inconsistency was also confirmed in a recent meta-analysis (Mahon, Yarcheski, Yarcheski, Cannella, & Hanks, 2006). In the general population, loneliness is more prevalent among adults without partners (Beutel et al., 2017), and also students living alone report more loneliness compared to those living in dorms or with a partner/friend (Diehl et al., 2018). Still, the literature remains sparse on the issue of identifying risk indicators of loneliness among young adults.

It has been suggested that the prevalence of loneliness is increasing, but very few studies have examined this over time (Cacioppo, Grippo, London, Goossens, & Cacioppo, 2015). A study of older Dutch people showed no change in loneliness from 2005 to 2010, with the exception of a subgroup of individuals with activity limitations, where the trend was increasing (Honigh-de Vlaming, Haveman-Nies, Groeniger, de Groot, & van ’t Veer, 2014). A similar stable pattern was observed in a Swedish study of an elderly population (Dahlberg, Agahi, & Lennartsson, 2018). In contrast, there was a rising rate of loneliness among Danish adolescents from 1991 to 2014, with the largest increase being observed among adolescents from families with high SES (Madsen et al., 2019).

This study addressed three main questions in a large nationally representative sample of young people: (1) what demographic factors are associated with loneliness in young adult college and university students? (2) How does partnership status offer protection from feelings of loneliness? and (3) Has the rate of loneliness changed from 2014 to 2018 in this population?

## Method

### Procedure

The SHoT study (*Students’ Health and Wellbeing Study)* is a national student survey for higher education in Norway. The main aim of the survey is to monitor students’ health, wellbeing and psychosocial environment. The survey has been carried out three times (2010, 2014 and 2018), and the two most recent waves (2014 and 2018) were used in the present study.

*The SHoT2014* study was conducted by the three largest student welfare organizations (*Sammen* [Bergen], *Sit* [Trondheim] and *SiO* [Oslo and Akershus]) in collaboration with, and with participation from, the 10 largest student welfare organizations in Norway, also targeting full-time Norwegian students < 35 years of age. Data for the *SHoT2014* study were collected electronically using a web-based platform in the period from 24 February 2014 to 27 March 2014. An invitation email containing a link to an anonymous online questionnaire was sent to 47,514 randomly selected students and stratified by study institutions, faculties, and departments. The overall response rate was 28.5% and included 13,525 students.

*The SHoT2018* was initiated by the three largest student welfare organizations (*Sammen* [Bergen and surrounding area], *Sit* [Trondheim and surrounding area] and *SiO* [Oslo and Akershus]), representing all student welfare organizations in Norway and done as a joint effort between these student welfare organisations and the Norwegian Institute of Public Health (NIPH). Data were collected between February 6 and April 5, 2018 and all fulltime Norwegian students aged between 18 and 35 years taking higher education (both in Norway and abroad) were invited to take part. The survey data were collected electronically through a web-based platform and some institutions allocated time during classes for the students to complete the set of questionnaires. For the SHoT2018 study, 162,512 students fulfilled the inclusion criteria, of whom 50,054 (30.8%) students completed the online questionnaires (Sivertsen, Råkil, Munkvik, & Lønning, 2019).

### Ethics

The authors assert that all procedures contributing to this work comply with the ethical standards of the relevant national and institutional committees on human experimentation and with the Helsinki Declaration of 1975, as revised in 2008. All procedures involving human subjects/patients were approved by the Regional Committee for Medical and Health Research Ethics in Western Norway (no. 2017/1176 [SHOT2018]). Informed consent was obtained electronically after the participants had received a detailed introduction to the study. Approvals for conducting the *SHoT2014* studies were granted by the Data Protection Officer for research at the Norwegian Centre for Research Data.

### Instruments

#### Demographic Information

All participants indicated their sex and age. In the current study, age was used both as a continuous and categorical variable, the latter employing the following age categories (18-20 years, 21-22 years, 23-25 years and 26-35 years). Participants were also asked about their relationship status (response options: “single”, “girl-/boyfriend”, “cohabitant”, and “married/ registered partner”), as well as their accommodation status (response options: “living alone”, “living with partner”, “living with friends/others in a collective”, and “living with parents”). Participants were categorized as an immigrant if either the student or his/her parents were born outside Norway. Finally, all students indicated if they were living or studying abroad.

#### Loneliness

Loneliness was measured by one item of the depression subscale of the HSCL-25 (Derogatis, Lipman, Rickels, Uhlenhuth, & Covi, 1974) in 2014 and 2018. *In the past two weeks, including today, how much have you been bothered by feeling lonely?* The response alternatives were “not at all”, “a little”, “quite a bit”, and “extremely”.

In SHoT2018 loneliness was assess using an abbreviated version of the widely used UCLA Loneliness Scale, “The Three-Item Loneliness Scale (T-ILS)” (Hughes, Waite, Hawkley, & Cacioppo, 2004). The T-ILS include the following three items, each rate along a 5-point Likert scale (“never”, “seldom”, “sometimes”, “often”, and “very often”). *For each question below, please indicate how often you have felt that way during the last year: 1) How often do you feel that you lack companionship? 2) How often do you feel left out, and 3) How often do you feel isolated from others?* The T-ILS has displayed satisfactory reliability and both concurrent and discriminant validity. (Hughes et al., 2004) In addition to analysing each of the three T-ILS items separately, we also calculated a total score, adding the three items together. The Cronbach’s alpha of the T-ILS total score was .88.

### Statistics

IBM SPSS version 25 (SPSS Inc., Chicago, IL, USA) for Mac was used for all analyses. Chi-square tests and logistic regression analysis were used to examine differences in the three loneliness items across demographical characteristics. Analysis of Variance (ANOVA) were conducted to examine potential polynomial/curvilinear associations between loneliness and age group by entering quadratic terms. We also used the Curve Estimation command in SPSS to test both the linear and curvilinear association between age as a continuous variable and overall loneliness. ANOVAs were also used to examine the T-ILS total score against the demographic variables. Effect sizes (pooled *SD*) were calculated using the Cohen’s *d* formula (Cohen, 1988). Pearson’s chi-squared tests were used to test for significant changes in loneliness over time. Missing values were handled using listwise deletion.

## Results

### Descriptive Characteristics

Compared to all invited students – 58.1% women (*n* = 93,267) and 41.9% men (*n* = 67,558) – the current sample included a larger proportion of women (69.1%) than men (30.9%). The mean age was 23.2 (*SD* = 3.3).

### Loneliness in SHoT2018

**Figure 1:**
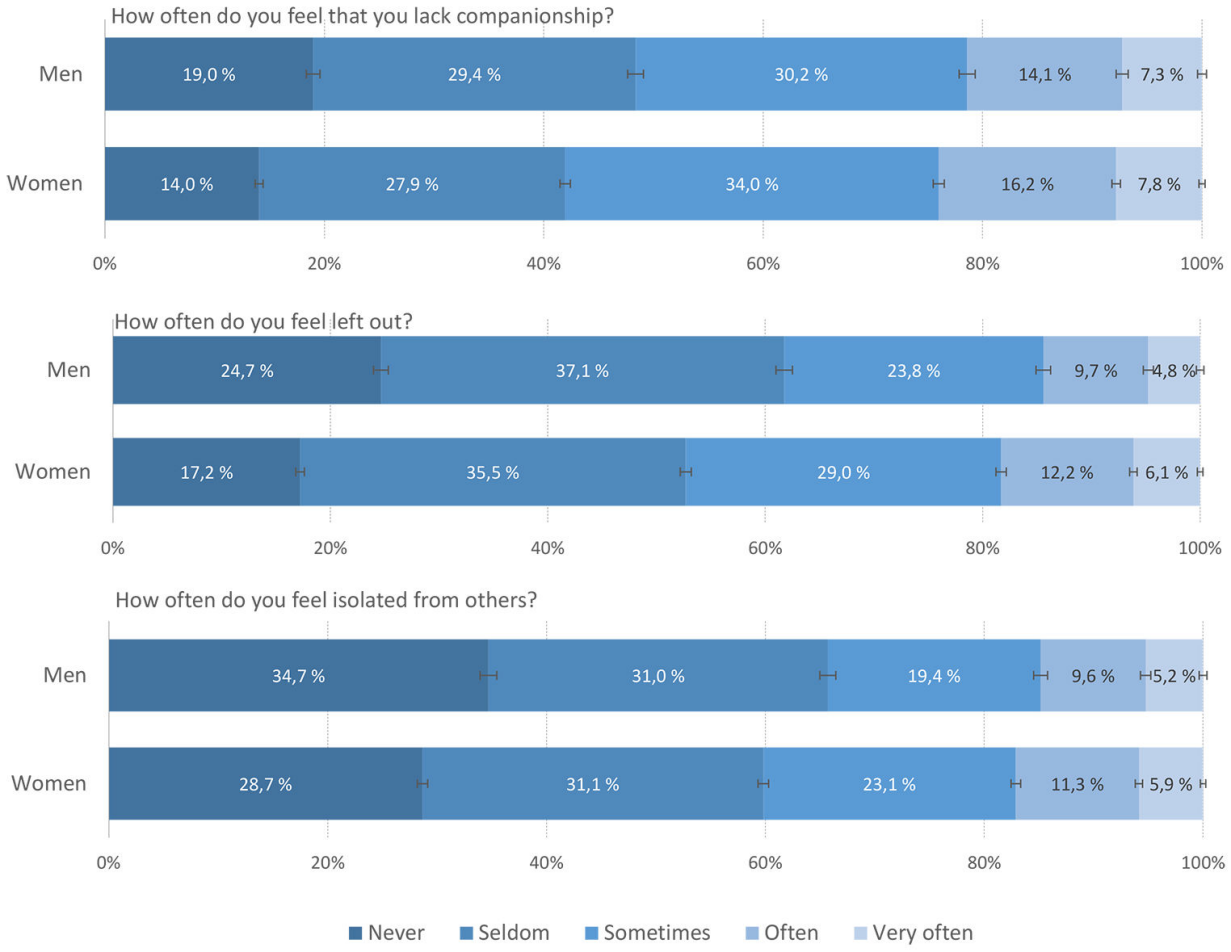
Response Pattern of the Three Loneliness Items in the T-ILS Among College/University Students in the SHoT2018 Study *Note.* Error bars represent 95% confidence intervals.

**Figure 2:**
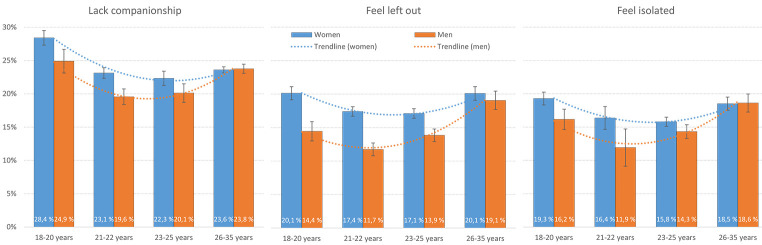
Loneliness Prevalence (“Often”/”Very Often”) Stratified by Age-Group in Male and Female Students *Note.* The curves show the polynomial/curvilinear trendline (order 2).

The response patterns of the three loneliness items are detailed in Figure 1. Almost one in four students (21% in males and 24% in females) felt that they lacked companionship “often” or “very often”. The corresponding estimates for the items “feeling left out” and “feeling isolated” were slightly lower, with 14%-15% in women and 17-18% in men (see Figure 2 for details). One in ten students (10.1%) reporting “often/very often” on all three items (females: 10.6% and males: 8.8%). All sex differences were statistically significant (*p* < .001).

### Loneliness and Age

Figure 2 shows the prevalence of the three loneliness items across the different age groups. As indicated by the dotted trend lines, there was a significant curvilinear relationship (all *p*s < .001) on all forms of loneliness for both men and women; both the youngest and oldest age-groups reported higher levels of both lacking companionships, feeling left out and feeling isolated (see Figure 2 for details). Table 1 shows the results from the logistic regression analyses. For example, compared to being 23-25 years old, female students aged between 18 and 20 years had 1.38 higher *OR*, 95% CI [1.29, 1.48], of reporting that they lacked companionship. There were significant sex × age interactions for all three loneliness items (see Table 1 for more details). As detailed in Table 2, analysing the T-ILS total score continuously showed a similar pattern U-shaped, with small Cohen’s *d* effect-sizes.

**Table 1:** Odds-Ratios (ORs) of Demographic Factors Associated With Loneliness (“Often” or “Very Often”) Among Norwegian University Students

Demographic factor	Lack companionship				Left out				Isolated			
	Women		Men		Women		Men		Women		Men	
	*OR*	95% CI	*OR*	95% CI	*OR*	95% CI	*OR*	95% CI	*OR*	95% CI	*OR*	95% CI
Age group	Sex interaction: Wald (*df*) = 11.41(3), *p* = .010				Sex interaction: Wald (*df*) = 29.70(3), *p* < .001				Sex interaction: Wald (*df*) = 25.28(3), *p* < .001			
18-20 years	1.38***	1.29, 1.48	1.32***	1.17, 1.48	1.22***	1.13, 1.32	1.05	0.91, 1.21	1.27***	1.18, 1.38	1.15*	1.01, 1.32
21-22 years	1.05	0.98, 1.12	0.97	0.87, 1.07	1.02	0.95, 1.10	0.82**	0.73, 0.93	1.04	0.97, 1.12	0.81***	0.72, 0.91
23-25 years	1.00		1.00		1.00		1.00		1.00		1.00	
26-35 years	1.07*	1.00, 1.16	1.24***	1.11, 1.38	1.22***	1.12, 1.32	1.47***	1.30, 1.65	1.21***	1.11, 1.32	1.37***	1.22, 1.54
Relationship status	Sex interaction: Wald (*df*) =159.58(3), *p* < .001				Sex interaction: Wald (*df*) = 88.48(3), *p* < .001				Sex interaction: Wald (*df*) = 82.32(3), *p* < .001			
Single	1.61***	1.39, 1.86	2.85***	2.14, 3.81	1.01	0.74, 1.18	1.59**	1.18, 2.14	1.18*	1.01, 1.39	1.65***	1.23, 2.21
Boy-/girlfriend	1.27**	1.09, 1.48	1.24	0.92, 1.68	1.02	0.74, 1.19	0.90	0.66, 1.22	1.05	0.90, 1.24	0.85	0.62, 1.16
Cohabitant	1.05	0.90, 1.22	0.91	0.67, 1.25	1.10	0.94, 1.29	1.08	0.79, 1.48	1.10	0.93, 1.29	0.94	0.69, 1.28
Married / registered partner	1.00		1.00		1.00		1.00		1.00		1.00	
Accommodation status	Sex interaction: Wald (*df*) = 86.75(3), *p* < .001				Sex interaction: Wald (*df*) = 32.32(3), *p* < .001				Sex interaction: Wald (*df*) = 36.86(3), *p* < .001			
Alone	1.96***	1.82, 2.11	3.85***	3.37, 4.39	1.26***	1.17, 1.37	1.99***	1.74, 2.28	1.50***	1.39, 1.62	2.44***	2.13, 2.80
With partner	1.00		1.00		1.00		1.00		1.00		1.00	
With friends / others in a collective	1.24***	1.16, 1.32	2.08***	1.85, 2.35	0.79***	0.74, 0.84	1.02	0.90, 1.15	0.85	0.80, 0.92	1.15*	1.02, 1.31
With parents	1.45***	1.31, 1.59	2.74***	2.33, 3.34	1.18**	1.07, 1.31	1.57***	1.32, 1.88	1.35***	1.21, 1.49	1.75***	1.46, 2.09
Immigration status	Sex interaction: Wald (*df*) = 3.77(1), *p* = .052				Sex interaction: Wald (*df*) = 0.16(1), *p* = .688				Sex interaction: Wald (*df*) = 1.65(1), *p* = .422			
Norwegian	1.00		1.00		1.00		1.00		1.00		1.00	
Immigrant	1.48***	1.35, 1.61	1.72***	1.51, 1.95	1.45***	1.32, 1.59	1.40***	1.20, 1.62	1.44***	1.31, 1.59	1.55***	1.34, 1.79
Studying abroad	Sex interaction: Wald (*df*) = 1.26(1), *p* = .262				Sex interaction: Wald (*df*) = 6.14(1), *p* = .013				Sex interaction: Wald (*df*) = 2.52(1), *p* = .112			
No	1.00		1.00		1.00		1.00		1.00		1.00	
Yes	1.19**	1.06, 1.35	1.04	0.84, 1.28	1.16*	1.02, 1.33	0.80	0.61, 1.04	1.16*	1.01, 1.33	0.92	0.71, 1.18

**Table 2:** Demographic Factors Associated With Loneliness (T-ILS Sum Score) Among Norwegian University Students

Demographic factor	Women			Men		
	*M*	*SD*	Cohen's *d^a^*	*M*	*SD*	Cohen's *d*
Age group						
18-20 years	7.90	3.11	0.12	7.32	3.10	0.15
21-22 years	7.60	3.00	0.02	6.87	2.93	Reference
23-25 years	7.55	2.98	Reference	7.07	3.02	0.07
26-35 years	7.66	3.19	0.04	7.50	3.26	0.20
Relationship status						
Single	7.78	3.05	0.17	7.62	3.15	0.36
Boy-/girlfriend	7.58	3.00	0.10	6.53	2.78	0.01
Cohabitant	7.52	3.08	0.08	6.52	2.88	0.01
Married / registered partner	7.27	3.07	Reference	6.49	3.04	Reference
Accommodation status						
Alone	8.25	3.16	0.25	8.09	3.26	0.52
With partner	7.49	3.07	0.01	6.48	(2.91)	Reference
With friends / others in a collective	7.47	2.92	Reference	7.02	2.92	0.18
With parents	7.86	3.22	0.13	7.36	3.30	0.28
Immigration status						
Norwegian	7.61	3.03	Reference	7.08	3.05	Reference
Immigrant	8.17	3.19	0.14	7.73	3.24	0.05
Studying abroad						
No	7.63	3.05	Reference	7.13	3.07	Reference
Yes	8.03	2.99	0.13	7.24	2.84	0.04

^a^Cohen’s *d* effect sizes (pooled *SD*) were calculated for each demographic variable using the category with the lowest T-ILS score (sex specific) as the reference group.

When analyzing the association between age as a continuous variable and overall loneliness, similar findings were observed. There was a statistically significant quadratic (curvilinear) association between continuous age and overall loneliness, *F*(2, 48685) = 12.91, *p* < .001, but there was no evidence of a significant linear association, *F*(1, 48686) = 1.48, *p* = .224).

### Loneliness and Relationship Status

**Figure 3:**
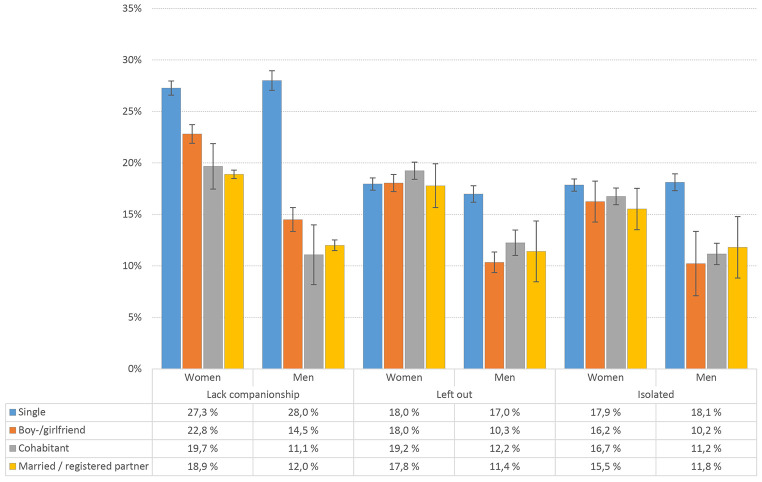
Loneliness Prevalence (“Often”/”Very Often”) by Relationship Status in Male and Female Students *Note.* Error bars represent 95% confidence intervals.

Single students reported more often that they *lacked companionship* compared to students with another relationship status, a tendency that was especially pronounced for single male students, *OR* = 2.85; 95% CI [2.14, 3.81], see Table 1 for details. And whereas *feeling left out* was also more prevalent in single male students, relationship status was not significantly associated with *feeling left out* in female students. In terms of feeling isolated, single male students reported higher levels of isolation, whereas relationship status was less clearly associated with feeling isolated in female students (see Figure 3 for details). There were significant sex × relationship interactions for all three loneliness items (all *p*s < .001). Analyses of the T-ILS total score showed a similar pattern (see Table 1 for details).

### Loneliness and Accommodation Status

**Figure 4:**
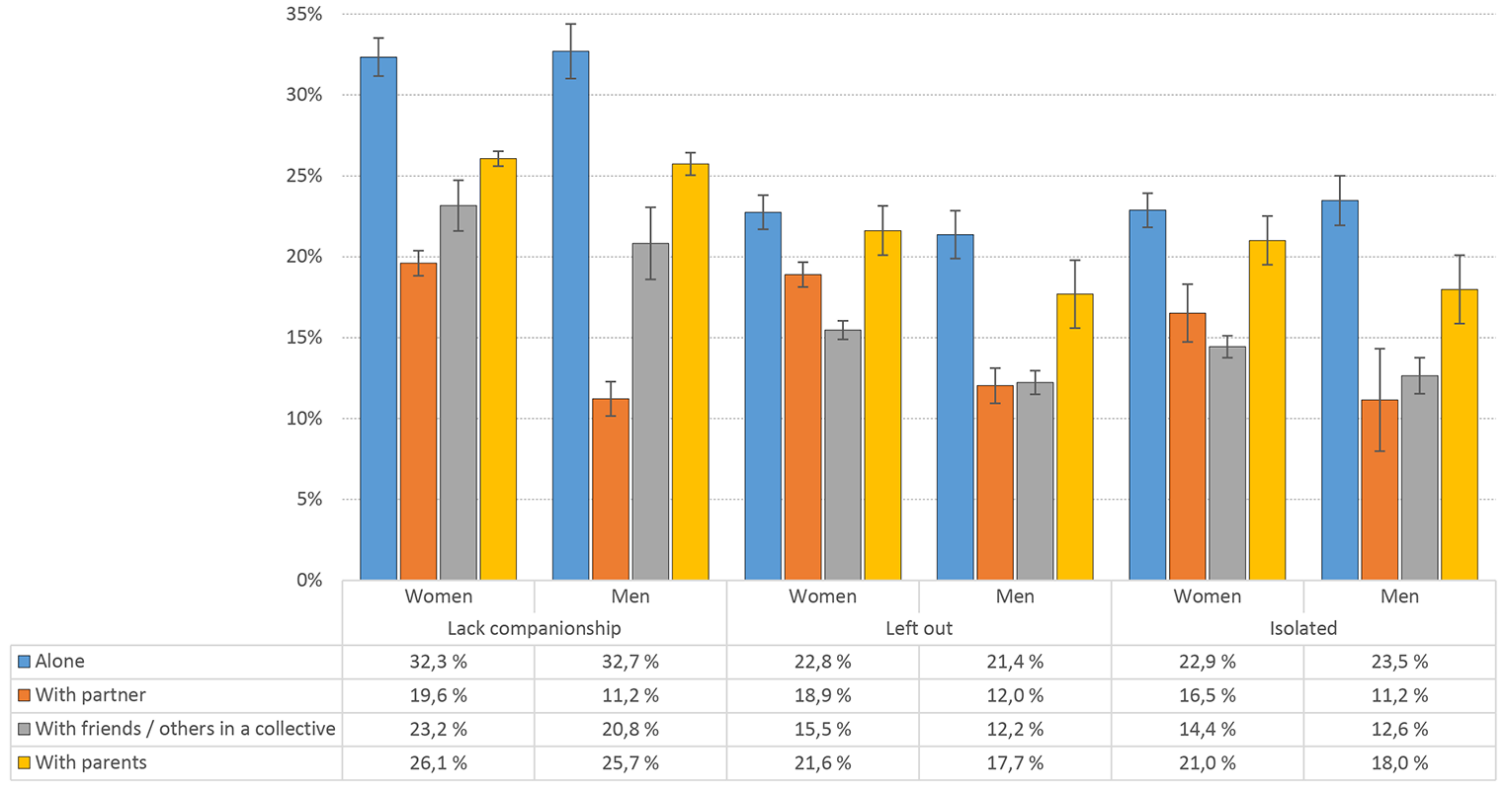
Loneliness Prevalence (“Often”/”Very Often”) by Accommodation Status in Male and Female Students *Note.* Error bars represent 95% confidence intervals.

Similar to the findings for relationship status, also accommodation status was significantly associated with loneliness. Both female and male, but especially male students living alone had the highest loneliness scores across all three items. Students living with their parents more often reported lacking companionship, feeling left out and isolated compared with students living with a partner/friends (see Figure 4 for details). There were significant sex × accommodation interactions for all three loneliness items (all *p*s < .001). Analyses of the T-ILS total score showed a similar pattern (see Table 1 for details).

### Loneliness and Studying Abroad

As detailed in Table 1, females students living/studing abroad had significantly higher odds of reporting loneliness across all three T-ILS items, whereas a similar pattern was not observed for male students. However, a significant sex × studying abroad interaction was only observed for “feeling left out” (see Table 1 for details).

### Trend of Loneliness From 2014 to 2018

**Figure 5:**
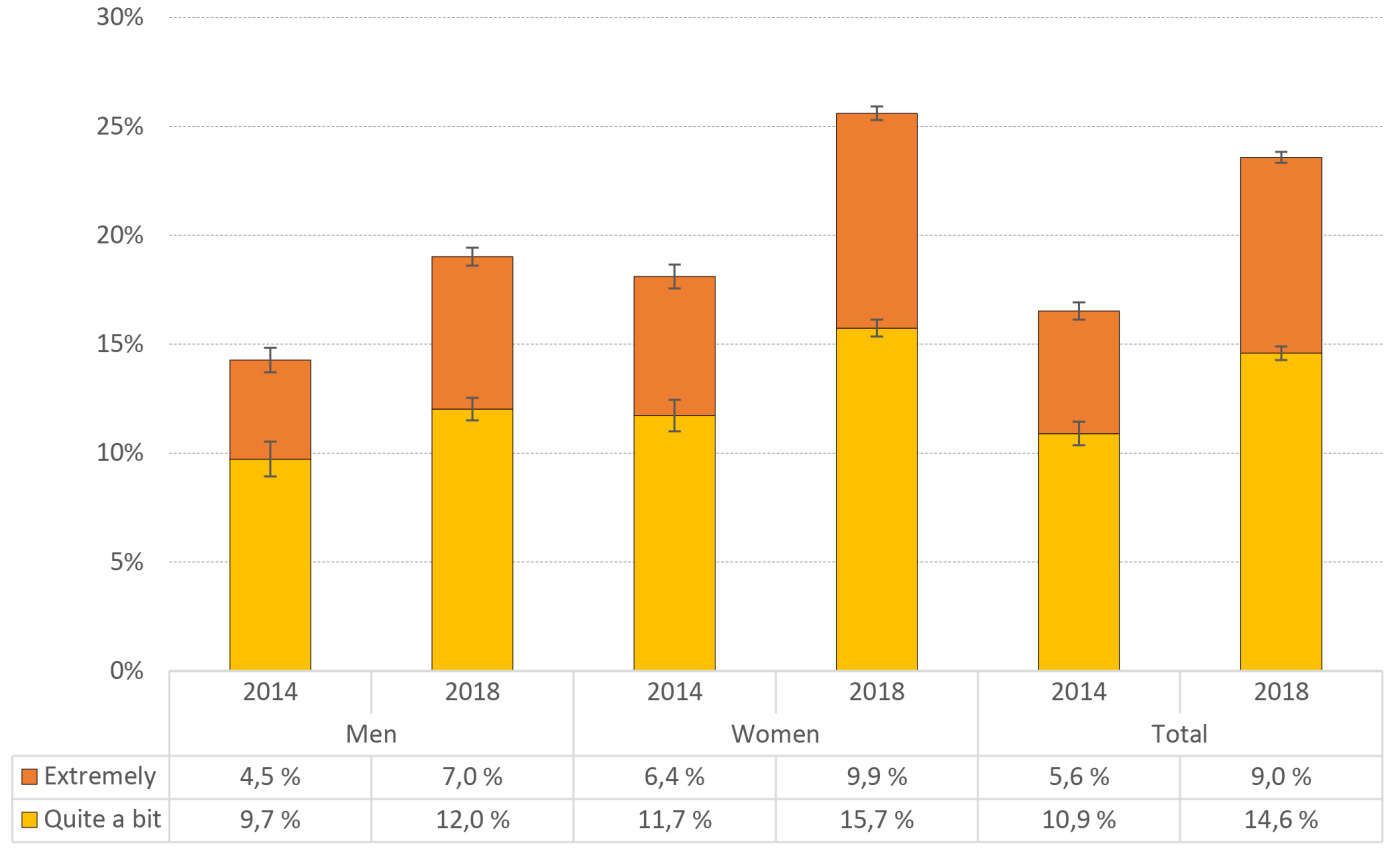
Prevalence of Loneliness (From the HSCL-25) From 2014 to 2018 by Sex *Note.* Error bars represent 95% confidence intervals.

Figure 5 shows the prevalence of loneliness across from 2014 to 2018. There was a significant overall increase in students reporting feeling lonely (“quite a bit”, or “extremely”) from 2014 (16.5%) to 2018 (23.6%; *p* < .001). The increase was evident in both men and women, and across both response categories (see Figure 5 for details).

## Discussion

This large national survey from 2018 of Norwegian fulltime students found that feelings of loneliness were common. Age showed a curvilinear association with loneliness, with the youngest and oldest students reporting the highest level of loneliness across all indicators of loneliness. Other significant demographic determinants of loneliness were being female, single, living alone and studying abroad. There was a considerable increase in loneliness reported by the 2018 cohort compared to the 2014 cohort, and this effect was particular strong for males, for whom the proportion of feeling “extremely” lonely had more than doubled.

The findings confirm that loneliness is frequently experienced among college and university students, as indicated by 14-24% of the students responding that they “often” or “very often” lacked companionship, felt left out, or felt isolated. In line with a previous German study (Diehl et al., 2018), we found that loneliness peaked among the youngest students (aged between 18-20 years), possibly as a result of the transition to university life. However, a second peak in loneliness was found among the oldest age group (26-35 years). This may be related to a second transitional period towards the end of the studies, in preparation for moving into full-time work. It may also be that these individuals are establishing new relationships after a transition into work and they identify less with student life and are spending less time in student social activities at this stage.

Being in a close relationships was associated with less loneliness among the students, comparable to the protective effect of close relationships in the general population (Beutel et al., 2017). For students, their living situation is a period-specific buffer against loneliness, with students sharing accommodation reporting less loneliness than those that live alone, a finding which also is in line with a previous German university study (Diehl et al., 2018).

The complex associations between sex and loneliness may be understood in light of the inconsistencies in sex differences in previous studies (Mahon et al., 2006). The general pattern is that female students report more loneliness than men across most categories, especially in the younger age groups, while the difference is attenuated in the oldest student group. Some risk factors had differential effects across sexes, including a stronger association between relationship status and loneliness among men, with single men being a noteworthy high-risk group. Similarly, living alone was also a stronger risk factor for men than women. Overall, it seems that men are more sensitive to the structural factors and relationship status for loneliness than females. This may also indicate that interventions should be attentive toward sex-specific risks, and it might be that differential interventions are needed. Future interventions studies could explore if men show a more beneficial more effect of structural interventions such as organised activites and housing, while women might respond better to strengthening social relationships. Women reporting more loneliness than men may also be a result of woman may more easily acknowledging feelings of loneliness, due to less social consequences of lonliness for woman (Borys & Perlman, 1985).

We found a substantial increase in reported loneliness from 2014 to 2018. While there is limited studies reporting on trends, a Danish study found a similar pattern from 1991 to 2014 among adolescents (Madsen et al., 2019). The effect in that study was strongest for the high SES groups. Although we have no information on family SES in the current study, all the included participants are pursuing higher education. Two studies of elderly have reported an opposite pattern, with loneliness decreasing over time (Honigh-de Vlaming et al., 2014; Lempinen, Junttila, & Sourander, 2018), but it might very well be that the trends are different across age groups, and this limits the comparison. The recency of the present study also precludes comparison to others in the same time period. It is uncertain if this is an ongoing trends, but the next planned wave of the SHoT study in 2022 will give new and valuable information on the longer trajectories of loneliness over time. What the drivers of this increase may be is also uncertain. It may reflect a general increase in mental distress, with recent evidence from the same dataset as the current study showing that both sleep problems and self-harm have increased across the same time period (Sivertsen, Hysing, et al., 2019; Sivertsen, Vedaa, et al., 2019).

The generalizabilty of the findings to the whole student population should be done with care given the relatively modest response rate for the SHoT2014 (29%) and SHoT2018 (31%). In relation to this, the issue of sample comparability is important. As the surveys in 2014 and 2018 included somewhat different welfare organizations and institutions, a recent report using the same datasets, performed detailed sensitivity analyses of the HSCL-25, comprising only institutions that were included in both surveys (Knapstad et al., 2019). The results from these analyses showed near-identical effect-sizes of the trend data, suggesting that the two samples from 2014 and 2018 are comparable.

Regarding the representativeness of the sample in comparison to the total student population in Norway, the SHoT2018 study consisted of 69% females, compared to 58% of all those who were invited. As such, this may represent a bias for the overall estimates, which is why we mainly present gender-specific results. In contrast, the age distribution was almost identical between the invited and the participating student, thus supporting the representativeness of the sample (Sivertsen, Vedaa, et al., 2019). Rather, it may be more appropriate to emphasize the relative differences between men and women, as well as different age cohorts and sociodemographic factors found in the current study, as these estimates are less prone to selection bias. The cross-sectional nature of the SHoT2018-study precludes conclusions on temporal order and causality. For instance, being lonely might reduce the chances for cohabiting, and thus loneliness might be a predictor of accomodation status and not its consequence. The loneliness measure is a three item, psychometrically sound measure, but a more nuanced understanding could have been gained by a more thorough assessment.

Future studies should investigate risk and protective factors for loneliness over and beyond demographic characteristics. Both individual characteristics of the students as well as systemic characteristics of the teaching situation should be investigated to increase our understanding of what constitutes risks for loneliness in this group to inform preventive interventions. The digital society may be one aspect that could account for the increase in loneliness and should be investigated further (Odacı & Kalkan, 2010) Further, if the trend of increasing loneliness will further strengthen in the coming years or will attenuate should be investigated in the present study as well as other epidemiological studies of students

The findings also have notable implications. The rise in loneliness over a four year period warrants concern, and should be met with preventive actions. The demographic determinants identified in this study could give indications of high risk groups to target, including the transitional periods and those living alone. There might be a need for interventions to target male and female students diffentially.
